# Pharmacoepidemiology and costs of medications dispensed during pregnancy: A retrospective population‐based study

**DOI:** 10.1111/1471-0528.17472

**Published:** 2023-04-11

**Authors:** Hannah Jackson, Luke E. Grzeskowiak, Joanne Enticott, Emily Callander

**Affiliations:** ^1^ Monash Centre for Health Research and Implementation (MCHRI), School of Public health and Preventive Medicine Monash University Clayton Victoria Australia; ^2^ College of Medicine and Public Health Flinders University Adelaide South Australia Australia; ^3^ SAHMRI Women and Kids South Australian Health and Medical Research Institute Adelaide South Australia Australia

**Keywords:** costs and cost analysis, dataset, demography, drugs, fetal, medication, obstetric, pharmaceutical preparations, surveillance

## Abstract

**Objective:**

To describe the pharmacoepidemiology and costs associated with medications dispensed during pregnancy.

**Design:**

Pharmacoepidemiological study and cost analysis.

**Setting:**

Queensland, Australia.

**Population:**

All women who gave birth in Queensland between January 2013 and June 2018.

**Methods:**

We used a whole‐of‐population linked administrative dataset, Maternity1000, to describe medications approved for public subsidy that were dispensed to 255 408 pregnant women. We describe the volume of medications dispensed and their associated costs from a Government and patient perspective.

**Main outcome measures:**

Prevalence of medication use; proportion of total dispensings; total medication costs in AUD 2020/21 ($1AUD = $0.67USD/£0.55GBP in December 2022).

**Results:**

During pregnancy, 61% (95% CI 60.96–61.29%) of women were dispensed at least one medication approved for public subsidy. The mean number of items dispensed per pregnancy increased from 2.14 (95% CI 2.11–2.17) in 2013 to 2.47 (95% CI 2.44–2.51) in 2017; an increase of 15%. Furthermore, mean Government cost per dispensing increased by 41% from $21.60 (95% CI $20.99–$22.20) in 2013 to $30.44 (95% CI $29.38–$31.49) in 2017. These factors influenced the 53% increase in total Government expenditure observed for medication use during pregnancy between 2013 and 2017 ($2,834,227 versus $4,324,377); a disproportionate rise compared with the 17% rise in women's total out‐of‐pocket expenses observed over the same timeframe ($1,880,961 versus $2,204,415).

**Conclusions:**

Prevalence of medication use in pregnancy is rising and is associated with disproportionate and rapidly escalating cost implications for the Government.

## 1 INTRODUCTION

Evidence suggests >80% of pregnant women take at least one medication during their pregnancy^1^, ^2^, ^3^, ^4^ and this prevalence has increased over time, with the average number of medicines used during a pregnancy increasing from 2.5 in 1976–1978 to 4.2 in 2006–2008.^4^ As the average age of women at childbirth rises,^5^ the incidence of maternal chronic disease is also rising.^6^, ^7^, ^8^ Some women enter pregnancy with chronic medical conditions that require ongoing or episodic pharmacological treatment (e.g. asthma, epilepsy, depression) and other women develop conditions during pregnancy that may require pharmaceutical intervention (e.g. iron deficiency anaemia, diabetes, pre‐eclampsia). Prior studies reporting on the epidemiology of pharmaceuticals used in pregnant women have largely focused on European cohorts,^9^, ^10^, ^11^, ^12^, ^13^, ^14^ although analyses have also been published on women in the UK,^15^ Australia,^16^, ^17^ North America,^4^, ^18^, ^19^ and Brazil,^2^, ^20^, ^21^ and one multinational study was also published in 2014.^1^ Of these 16 studies, only seven (44%) were published in the last decade and only one study^2^ reported on the costs associated with the medications prescribed.

Healthcare budgets are finite and the resources available to achieve positive health outcomes are limited, therefore even when there are data to support the safety, efficacy and economic efficiency of medication use (known information deficits for pregnant populations^22^, ^23^, ^24^, ^25^, ^26^, ^27^, ^28^), due consideration must also be given to the opportunity cost (the value foregone as a consequence of a resource not being available for its best alternative use) associated with implementation at scale.^23^ Increasing the proportion of Government budgets that are spent on healthcare reduces the proportion available for other societal priorities such as education, housing and transportation. Rising healthcare costs can impact the economy, compromise patient care and financial security, and impact patient access to care.^29^ Evidence of the affordability, cost and value of healthcare interventions is essential to inform national health priorities and support the development of clinical practice guidelines.

The objective of this study is to give an overview of the pharmacoepidemiology and costs of prescription medications dispensed during pregnancy among a cohort of Australian women, using a rich source of routinely collected health information. More specifically, we aim to describe the prevalence of medication use during pregnancy and identify which medications are the greatest cost‐drivers for total expenditure from the perspective of both women and the Government.

## 2 METHODS

### 2.1 Study design and population

This population‐level observational study utilises an existing linked administrative dataset, Maternity1000,^30^ which contains information on 255 408 women (328 868 pregnancies) in Queensland, Australia, who gave birth between 1 January 2013 and 30 June 2018 and their infants. The data as of June 2018 were the latest data that could be released by government data custodians to the research team. For the study described in this paper, routinely collected information on all pregnant women and their infants born (live births and stillbirths of ≥20 weeks’ gestation or ≥400 g) during this timeframe were identified from the Queensland Perinatal Data Collection (PDC) and records for mothers and children were then linked to Pharmaceutical Benefits Scheme (PBS) claims & costs records between 1 September 2011 and 30 June 2018. Only prescriptions that were dispensed under the funding provisions of the Pharmaceutical Benefits Scheme were included. Data were available for *all* medicines dispensed during the antenatal period (i.e. including under co‐payment dispensings) for this population.^31^ Births in all sectors (i.e. public and private) were included in the analysis, with equal access to PBS‐subsidised prescriptions for public and private patients provided they are entitled to Medicare benefits. Patients are eligible for Medicare if they are an Australian or New Zealand Citizen, an Australian permanent resident, have applied for permanent residency, are a temporary resident covered by a ministerial order, or are visiting from a country with a Reciprocal Health Care Agreement.^32^ In Australia, medicines listed on the PBS can be dispensed to patients at a Commonwealth Government subsidised price. There were 906 different medicines listed on the PBS as of 30 June 2021.^33^ Patients pay a co‐payment towards the cost of each PBS‐subsidised medicine. In 2022, general patients pay up to $42.50 per item dispensed and concession card holders pay up to $6.80 per item.^34^ Births with an unknown birth year (*n* = 93) were excluded from our analysis.

Prescription drug use in pregnancy was defined for this analysis as the dispensing of any PBS‐listed medication (i.e. a medication approved for public subsidy) to a woman after day 30 of pregnancy up until the date of delivery. A diagram showing the variables we used to define a dispensing that occurred during pregnancy within our dataset is shown in Figure S1. We excluded the first 30 days of pregnancy to avoid misclassification of medications potentially dispensed in the month prior to pregnancy.

Neither patients nor the public were involved in the development of the dataset utilised for this study, or in the design of the analyses (see Table S1 for GRIPP2‐SF checklist). We acknowledge the value that such engagement can divulge, but we were unable to integrate this into our study. In addition, we were unable to incorporate a core outcome set, as we primarily focus on dispensing data and associated cost analyses, rather than health outcomes.

### 2.2 Statistical analysis

Using the information contained within the Maternity1000 dataset, a pharmacoepidemiological analysis^35^ was conducted on the use of pharmaceuticals dispensed during pregnancy. The unit of analysis for demographic data was one pregnancy; therefore women may appear in the results more than once if they had multiple pregnancies during the timeframe analysed. We defined the first trimester as covering the period from 31 days up to 13^+6^ weeks’ gestation; second trimester as 14^+0^ weeks to 27^+6^ weeks’ gestation; and third trimester as 28^+0^ weeks until the date of delivery. This definition is aligned with that used by The American College of Obstetricians and Gynecologists.^36^

Statistical analysis was conducted using SAS Version 9.4. We used descriptive statistics to illustrate:

- the prevalence of pregnant women dispensed ≥1 PBS‐listed pharmaceutical throughout:

1. each trimester of pregnancy, and
2. the entire pregnancy;

- the most frequently dispensed medications (reported as the number of dispensings for a given medication as a percentage of the total number of dispensings for all medications) in terms of:

1. pharmaceutical agents and
2. the World Health Organization's Anatomical Therapeutic Chemical (ATC) Classification System^37^;

- which medications represent the greatest cost burden over the study period for:

1. women (through out‐of‐pocket (OOP) payments) and
2. the Government (PBS subsidy amount).

Total medication costs can be calculated by adding patient costs (OOP payments) to Government costs (PBS subsidy amount), therefore we have not explicitly presented or discussed these in our analyses. Normal distributions were assumed for all cost and count data, with 95% CI presented for all mean values. Standard Wald confidence limits for proportions were calculated for 95% CI reported for the prevalence of women dispensed ≥1 PBS‐listed medication during pregnancy.

We conducted a sub‐group analysis to investigate whether there were any distributional effects according to the mother's charging status (i.e. the type of ward accommodation under which the mother elected to be admitted) – colloquially referred to as a public admission (universal healthcare often fully paid by the government) and private admission (combination of fees including private fees paid for by the patient).

Within the dataset, date of delivery is only available as the month and year of birth, with the date always listed as the first of the month in an effort to maintain privacy. Sensitivity analyses were conducted to test the robustness of our results to different assumptions regarding date of delivery. We tested two alternate scenarios: the assumed date of birth being the end of the month and the assumed date of birth being the 15th of the month displayed in the dataset. The outcomes tested in this analysis were the most frequently dispensed medications and the medications that were the greatest cost‐burden for the Government. Kendall's coefficient of concordance was calculated to determine whether rankings across the three scenarios differed significantly.

Where total annual costs are calculated, data are only presented graphically for women who gave birth from 2013 up until the end of 2017 to ensure a consistent balance in the number of pregnancies reported across the years, as a full calendar year of data was not available for 2018. Therefore, the costs of medications presented for the year 2013 refer to the total cost of any PBS items dispensed during pregnancy to a woman who gave birth in 2013 (i.e. not simply the items dispensed during the 2013 calendar year). All cost data have been adjusted for inflation using the Reserve Bank of Australia's Inflation Calculator^38^ and are presented in constant prices; 2020/21 Australian Dollars ($1AUD = $0.67USD/£0.55GBP in December 2022).

## 3 RESULTS

### 3.1 Demographics

Demographic characteristics for women in the dataset are presented in Table 1. More than 98% of pregnancies were singleton pregnancies, and 31% of all pregnancies were first‐time pregnancies. Almost 30% of women had a medical condition diagnosed prior to or during pregnancy, with 20% of women entering pregnancy with obesity. In addition, 70% of women experienced a complication during their pregnancy. Trends in demographic characteristics over time show the mean age of pregnant women, mean body mass index (BMI) at conception and the percentage of pregnant women with a diagnosed medical condition or experiencing a complication during pregnancy have increased from 2013 to 2018 (see Figure S2). A modified version of Table 1 is also reported in Table S2, which incorporates the numbers and percentage of missing data for each variable.

**TABLE 1:** Characteristics of pregnant women, Queensland, Australia, 2013–2018^a^.

Maternal characteristics	Number (*n*)	Percentage (%)
Plurality		
Singleton	323 912	98.49
Multiple	4956	1.51
Maternal age		
<20	9151	2.78
20 to <35	245 202	74.56
≥35	74 515	22.66
Gravidity		
First pregnancy (primigravida)	100 568	30.58
Not first pregnancy (multigravida)	228 297	69.42
Country of birth		
Australia	240 515	73.14
Other	88 336	26.86
Indigenous status		
Yes	19 231	5.85
No	309 623	94.15
Funding for antenatal care		
Public	226 817	69.11
Private	101 372	30.89
Smoking status		
Before 20 weeks		
Yes	38 865	11.85
No	289 044	88.15
After 20 weeks		
Yes	31 396	9.59
No	296 042	90.41
Body mass index (BMI) category^b^		
Underweight	18 196	5.61
Healthy weight	168 251	51.87
Overweight	74 210	22.88
Obese	63 700	19.64
Medical conditions^c^		
Yes	97 403	29.62
No	231 457	70.38
Pregnancy complication^d^		
Yes	230 200	70.00
No	98 661	30.00
Assisted reproductive technology (ART)		
Yes	17 371	5.28
No	311 489	94.72
Born alive		
Yes	327 278	99.52
No (stillbirth)	1590	0.48
Total number of pregnancies^e^	**328 868**	**100**

^a^ Data only available for births up until 30 June 2018.

^b^ BMI calculation uses the self‐reported weight of the mother in the 4–6 weeks prior to or at conception.^46^

^c^ Pre‐existing maternal conditions, diseases or illnesses (e.g. hypertension, diabetes) and other conditions, diseases or illnesses that arise during the current pregnancy. Conditions included here are not directly caused by the pregnancy but may influence pregnancy care.^46^

^d^ Complications of pregnancy that arise prior to the initiation of labour or birth. Conditions included here are directly caused by the pregnancy and may influence pregnancy care.^46^

^e^ Sum of observations for each category does not always equal the total due to missing data for some variables.

### 3.2 Prevalence of at least one medication approved for public subsidy being dispensed during pregnancy

Across the analysis period (2013–2018), 61% (95% CI 60.96–61.29) of pregnant women were dispensed ≥1 PBS‐listed medication during pregnancy (see Table S3). Prevalence increased over the timeframe investigated and was marginally higher during the first trimester than during the second or third trimester.

### 3.3 Medications dispensed in the greatest volume during pregnancy

Table 2 shows that metoclopramide (11%), amoxicillin (10%) and cefalexin (9%) were the three most commonly dispensed medications. Antibacterials for systemic use were the most frequently dispensed therapeutic class, making up 26% of all mediations dispensed. Psychoanaleptics were the next most common therapeutic class at just over 11% of all dispensings, closely followed by drugs for functional gastrointestinal disorders (11%), which incorporates metoclopramide dispensings.

**TABLE 2:** The 20 most frequently dispensed pharmaceutical agents (as percentage of total prescriptions dispensed to pregnant women) and therapeutic classes (using the 2nd level of the Anatomical Therapeutic Chemical (ATC) Classification System) during pregnancy, Queensland, Australia, 2013–2018^a^.

Most frequently dispensed pharmaceutical agents					Most frequently dispensed therapeutic classes				
Pharmaceutical agent (ATC code)	Frequency	Percent	Cumulative percent	Prevalence^b^ (%)	ATC classification (ATC code, 2nd level)	Frequency	Percent	Cumulative percent	Prevalence^b^ (%)
1. Metoclopramide (A03FA01)	84 504	11.04	11.04	18.18	1. Antibacterials for systemic use (J01)	199 496	26.05	26.05	34.51
2. Amoxicillin (J01CA04)	72 889	9.52	20.55	16.62	2. Psychoanaleptics (N06)	86 597	11.31	37.36	5.77
3. Cefalexin (J01DB01)	72 664	9.49	30.04	15.85	3. Drugs for functional gastrointestinal disorders (A03)	84 982	11.10	48.46	18.25
4. Sertraline (N06AB06)	25 380	3.31	33.36	1.86	4. Drugs for acid‐related disorders (A02)	49 977	6.53	54.98	7.00
5. Paracetamol + codeine (N02AJ06)	24 438	3.19	36.55	5.13	5. Analgesics (N02)	44 124	5.76	60.75	6.97
6. Metformin (A10BA02)	23 429	3.06	39.61	3.97	6. Drugs for obstructive airway diseases (R03)	41 071	5.36	66.11	5.68
7. Ranitidine (A02BA02)	21 919	2.86	42.47	3.92	7. Drugs used in diabetes (A10)	39 250	5.13	71.24	5.42
8. Enoxaparin sodium (B01AB05)	20 864	2.72	45.20	1.44	8. Antianaemic preparations (B03)	22 789	2.98	74.21	5.64
9. Levothyroxine (H03AA01)	20 040	2.62	47.81	3.86	9. Antiemetics and antinauseants (A04)	22 418	2.93	77.14	3.79
10. Ondansetron (A04AA01)	19 986	2.61	50.42	3.34	10. Antithrombotic agents (B01)	22 043	2.88	80.02	1.57
11. Salbutamol (R03AC02)	18 427	2.41	52.83	3.44	11. Thyroid therapy (H03)	21 109	2.76	82.77	4.01
12. Escitalopram (N06AB10)	15 469	2.02	54.85	1.15	12. Sex hormones and modulators of the genital system (G03)	21 059	2.75	85.52	2.41
13. Amoxicillin + clavulanic acid (J01CR02)	13 282	1.73	56.58	3.11	13. Psycholeptics (N05)	15 831	2.07	87.59	1.91
14. Venlafaxine (N06AX16)	10 918	1.43	58.01	0.64	14. Corticosteroids, dermatological preparations (D07)	15 611	2.04	89.63	3.61
15. Rabeprazole (A02BC04)	10 722	1.40	59.41	1.37	15. Corticosteroids for systemic use (H02)	14 225	1.86	91.49	2.62
16. Fluticasone propionate + salmeterol (R03AK06)	9914	1.29	60.70	1.39	16. Antiepileptics (N03)	8704	1.14	92.63	0.39
17. Estradiol (G03CA03)	9598	1.25	61.96	0.91	17. Antihypertensives (C02)	8204	1.07	93.70	0.76
18. Erythromycin ethylsuccinate^c^ (J01FA01)	8627	1.13	63.08	2.03	18. Antivirals for systemic use (J05)	6385	0.83	94.53	1.07
19. Desvenlafaxine (N06AX23)	8447	1.10	64.19	0.59	19. Opthalmologicals (S01)	5193	0.68	95.21	1.18
20. Oxycodone (N02AA05)	8145	1.06	65.25	1.30	20. Beta blocking agents (C07)	5187	0.68	95.89	0.77

^a^ Data only available for births up until 30 June 2018.

^b^ A prevalent case is defined here as a woman who was dispensed a given medication at least once during her pregnancy.

^c^ NB: Erythromycin ethyl succinate in tablet form is no longer listed on the PBS; erythromycin 250mg enteric capsules are currently listed.

### 3.4 Medications that cost the most during pregnancy

**FIGURE 1:**
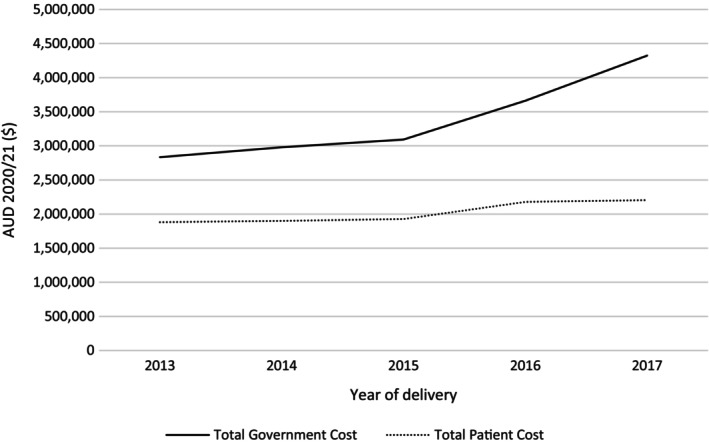
Total annual cost of PBS‐listed medications dispensed during pregnancy to both patients and the Government, 2013 to 2017, in constant prices (AUD 2020/21).

For women who gave birth in 2017, the Government spent more than $4.32 million (AUD 2020/21) on PBS‐listed medications dispensed during pregnancy (see Table 3). Total Government cost increased rapidly over the timeframe analysed. Total out‐of‐pocket expenses for pregnant women also increased over the period analysed, albeit at a slower rate (see Figure 1 for graphical representation of the results). This indicates a trend towards more expensive drugs (i.e. drugs with a total cost above the patient co‐payment) being dispensed over time.

**TABLE 3:** Cost of Pharmaceutical Benefits Scheme (PBS) listed medications dispensed during pregnancy to both patients and the Government over time, Queensland, Australia, 2013–2018^a^, in constant prices (AUD 2020/21).

Year of delivery	Number of dispensings	Number of women who gave birth	Mean dispensings per pregnancy (95% CI)	Government costs			Patient costs		
				Total Government expenditure	Mean Government cost per dispensing (95% CI)	Mean Government expenditure per pregnancy (95% CI)	Total patient OOP expenditure	Mean patient OOP cost per dispensing (95% CI)	Mean patient OOP expenditure per pregnancy (95% CI)
All PBS prescriptions									
2013	131 219	61 409	2.14 (2.11–2.17)	$2,834,227	$21.60 (20.99–22.20)	$46.15 (42.76–49.15)	$1,880,961	$14.33 (14.27–14.40)	$30.63 (30.08–31.18)
2014	136 035	61 043	2.23 (2.20–2.26)	$2,979,208	$21.90 (21.31–22.49)	$48.81 (45.64–51.97)	$1,900,249	$13.97 (13.91–14.03)	$31.13 (30.58–31.68)
2015	140 253	59 780	2.35 (2.31–2.38)	$3,092,140	$22.05 (21.37–22.72)	$51.73 (48.26–55.19)	$1,926,684	$13.74 (13.68–13.79)	$32.23 (31.67–32.79)
2016	143 532	59 987	2.39 (2.36–2.43)	$3,662,126	$25.51 (24.78–26.25)	$61.05 (57.41–64.68)	$2,177,747	$15.17 (15.12–15.23)	$36.30 (35.71–36.90)
2017	142 076	57 476	2.47 (2.44–2.51)	$4,324,377	$30.44 (29.38–31.49)	$75.24 (70.25–80.22)	$2,204,415	$15.52 (15.46–15.57)	$38.35 (37.70–39.01)
2018^a^	72 645	29 173	2.49 (2.44–2.54)	$2,438,957	$33.57 (31.39–35.75)	$83.60 (68.23–98.98)	$1,120,621	$15.43 (15.35–15.51)	$38.41 (37.55–39.28)
Subgroup analysis according to mother's hospital ward accommodation (public versus private)									
Public									
2013	88 308	41 002	2.15 (2.12–2.19)	$1,948,767	$22.07 (21.33–22.80)	$47.53 (43.41–51.65)	$1,047,170	$11.86 (11.79–11.92)	$25.54 (25.01–26.07)
2014	93 040	41 623	2.24 (2.20–2.27)	$2,045,073	$21.98 (21.28–22.68)	$49.13 (45.40–52.87)	$1,097,564	$11.80 (11.73–11.86)	$26.37 (25.83–26.91)
2015	95 715	41 248	2.32 (2.28–2.36)	$2,020,921	$21.11 (20.41–21.82)	$48.99 (45.39–52.60)	$1,115,327	$11.65 (11.59–11.71)	$27.04 (26.51–27.57)
2016	98 566	41 455	2.38 (2.34–2.42)	$2,346,063	$23.80 (22.94–24.67)	$56.59 (52.45–60.74)	$1,270,184	$12.89 (12.83–12.95)	$30.64 (30.06–31.22)
2017	97 494	40 427	2.41 (2.37–2.45)	$2,736,033	$28.06 (26.67–29.45)	$67.68 (61.49–73.86)	$1,277,273	$13.10 (13.04–13.16)	$31.59 (30.97–32.22)
2018^a^	51 868	21 062	2.46 (2.40–2.52)	$1,632,426	$31.47 (28.59–34.35)	$77.51 (56.69–98.33)	$693,360	$13.37 (13.28–13.45)	$32.92 (32.07–33.77)
Private									
2013	42 815	20 306	2.11 (2.06–2.16)	$884,187.22	$20.65 (19.58–21.72)	$43.54 (37.53–49.55)	$832,423	$19.44 (19.33–19.56)	$40.99 (39.73–42.26)
2014	42 920	19 324	2.22 (2.17–2.28)	$933,551.83	$21.75 (20.63–22.87)	$48.31 (42.38–54.25)	$801,774	$18.68 (18.57–18.80)	$41.49 (40.23–42.76)
2015	44 414	18 420	2.41 (2.35–2.47)	$1,069,893.45	$24.09 (22.60–25.58)	$58.08 (50.25–65.91)	$809,308	$18.22 (18.11–18.33)	$43.94 (42.57–45.30)
2016	44 809	18 372	2.44 (2.38–2.50)	$1,314,369.36	$29.33 (27.96–30.70)	$71.54 (64.23–78.85)	$905,106	$20.20 (20.09–20.30)	$49.27 (47.84–50.69)
2017	44 433	16 893	2.63 (2.56–2.70)	$1,578,949.96	$35.54 (34.08–36.99)	$93.47 (85.22–101.71)	$924,154	$20.80 (20.69–20.91)	$54.71 (53.10–56.31)
2018^a^	20 700	8057	2.57 (2.47–2.66)	$805,413.74	$38.91 (36.37–41.44)	$99.96 (88.25–111.68)	$425,850	$20.57 (20.42–20.72)	$52.85 (50.68–55.03)

Abbreviation: OOP, out‐of‐pocket.

^a^ Data only available for births up until 30 June 2018 (i.e. only 6 months of data for 2018).

In terms of individual medications, Table 4 shows that women spent the greatest amount of money on the antiemetic metoclopramide and the anticoagulant/antithrombotic agent enoxaparin sodium, which was also the medication that the Government spent the greatest amount of money on. Medications representing the highest out‐of‐pocket costs to women were approximately in line with volume of use, with 80% of the top 20 most frequently dispensed medications also being listed in the 20 highest total cost contributors for women's OOP expenditure. Antibacterials for systemic use and psychoanaleptics contributed both the greatest volume and cost to women in terms of therapeutic class (Tables 2 and S4).

**TABLE 4:** The 20 medications that contribute the greatest total cost to either pregnant women or the Government, Queensland, Australia, 2013–2018^a^, in constant prices (AUD 2020/21).

Total cost to pregnant women (total OOP expenditure)					Total Government expenditure (PBS expenditure)				
Pharmaceutical agent (ATC code)	Number of dispensings	Total patient expenditure per medication	Percent of total patient expenditure	Cumulative percent	Pharmaceutical agent (ATC code)	Number of dispensings	Government expenditure	Percent of total government expenditure	Cumulative percent
1. Metoclopramide (A03FA01)	84 504	$785,845.61	7.01	7.01	1. Enoxaparin sodium (B01AB05)	20 864	$2,581,818.81	13.36	13.36
2. Enoxaparin sodium (B01AB05)	20 864	$702,169.30	6.26	13.27	2. Ferric carboxymaltose (B03AC)	7301	$2,041,785.79	10.56	23.92
3. Amoxicillin (J01CA04)	72 889	$695,572.56	6.20	19.48	3. Insulin aspart (A10AB05)	5684	$1,155,155.55	5.98	29.89
4. Cefalexin (J01DB01)	72 664	$685,100.11	6.11	25.59	4. Adalimumab (L04AB04)	591	$1,076,963.84	5.57	35.46
5. Levothyroxine (H03AA01)	20 040	$481,321.17	4.29	29.88	5. Insulin isophane human (A10AC01)	5797	$785,529.93	4.06	39.53
6. Ondansetron (A04AA01)	19 986	$343,736.30	3.07	32.95	6. Insulin glargine (A10AE04)	1507	$585,211.54	3.03	42.56
7. Sertraline (N06AB06)	25 380	$302,147.84	2.70	35.64	7. Mesalazine (A07EC02)	2128	$572,384.95	2.96	45.52
8. Metformin (A10BA02)	23 429	$287,299.79	2.56	38.21	8. Insulin detemir (A10AE05)	1435	$543,545.13	2.81	48.33
9. Ranitidine (A02BA02)	21 919	$285,888.21	2.55	40.76	9. Infliximab (L04AB02)	177	$522,963.85	2.71	51.03
10. Fluticasone propionate + salmeterol (R03AK06)	9914	$272,414.13	2.43	43.19	10. Fluticasone propionate + salmeterol (R03AK06)	9914	$445,426.25	2.30	53.34
11. Ferric carboxymaltose (B03AC)	7301	$233,504.17	2.08	45.27	11. Etanercept (L04AB01)	239	$404,147.57	2.09	55.43
12. Rabeprazole (A02BC04)	10 722	$203,487.74	1.82	47.08	12. Tenofovir disoproxil (J05AF07)	353	$341,636.27	1.77	57.20
13. Paracetamol + codeine (N02AJ06)	24 438	$201,351.70	1.80	48.88	13. Follitropin alfa (G03GA05)	248	$337,385.50	1.75	58.94
14. Escitalopram (N06AB10)	15 469	$201,238.89	1.80	50.68	14. Amino acid formula with vitamins and minerals w/out Phe (V06DX)	140	$258,008.44	1.33	60.28
15. Desvenlafaxine (N06AX23)	8447	$198,581.62	1.77	52.45	15. Budesonide + formoterol (eformoterol) (R03AK07)	6069	$246,651.79	1.28	61.55
16. Budesonide + formoterol (eformoterol) (R03AK07)	6069	$182,446.56	1.63	54.07	16. Ivacaftor (R07AX02)	10	$237,949.12	1.23	62.78
17. Salbutamol (R03AC02)	18 427	$182,436.64	1.63	55.70	17. Progesterone (G03DA04)	1034	$206,098.80	1.07	63.85
18. Venlafaxine (N06AX16)	10 918	$176,998.08	1.58	57.28	18. Desvenlafaxine (N06AX23)	8447	$189,315.49	0.98	64.83
19. Medroxyprogesterone (G03DA02)	6228	$173,837.74	1.55	58.83	19. Quetiapine (N05AH04)	2832	$180,324.62	0.93	65.76
20. Insulin isophane human (A10AC01)	5797	$171,556.43	1.53	60.36	20. Valaciclovir (J05AB11)	3049	$168,395.56	0.87	66.63

Abbreviation: Phe, phenylalanine.

^a^ Data only available for births up until 30th June 2018.

Injectable agents featured heavily as high total cost items for the Government, with eight of the top ten pharmaceutical agents being injectables (see Table 4). In addition, insulin preparations accounted for four of the ten highest cost pharmaceuticals to the Government. Accordingly, antidiabetic therapies are the largest cost‐contributor to the Government in terms of therapeutic category (see Table S4). More than half of total Government expenditure on PBS‐listed pharmaceuticals for pregnant women were attributable to only nine pharmaceutical agents (see Table 4).

### 3.5 Medication expenditure during pregnancy for women electing to be admitted as a public versus private patient

Table 3 shows that Government expenditure per prescription and per pregnancy (on medication) is increasing at a more rapid rate for women who elect private obstetric care versus those who elect public obstetric care (refer to Figure S3 for graphical representation of results). This is despite a very modest increase in the mean number of PBS‐listed prescriptions dispensed per pregnancy for private versus public patients. The average patient out‐of‐pocket costs per pregnancy are far greater for women whose antenatal care is funded privately (private = $52.85 versus public = $32.92 in 2018), although this is influenced by a larger proportion of concession card holders being cared for publicly (private: general = 93% versus concession = 7%; public: general = 54% versus concession = 46%) and therefore a lower average patient contribution to the cost per dispensing, as shown in Table 3.

### 3.6 Sensitivity analyses

When the assumed date of delivery was altered to the 15th of the birth month rather than the 1st of the month supplied in the dataset, 18 of the 20 most frequently dispensed pharmaceutical agents during pregnancy in the primary analysis remained in the top 20. The same was true for the 20 medications that contributed the greatest total cost to Government expenditure, as shown in Tables S5 and S6. Modifying the assumption to the date of delivery being the end of the birth month showed that 17 of the 20 most frequently dispensed pharmaceutical agents remained in the top 20. In terms of total Government expenditure, 18 of the top 20 pharmaceuticals remained in the top 20. Calculation of Kendall's coefficient of concordance revealed significant agreement between the rankings shown in each scenario (frequency of dispensing: *W* = 0.90, *p* < 0.0001; total Government expenditure: *W* = 0.94, *p* < 0.0001). The sensitivity analyses therefore show the results are robust to reasonable changes in the assumed date of delivery.

## 4 DISCUSSION

### 4.1 Main findings

Overall, six in every ten pregnant women were dispensed at least one prescription medication during pregnancy and prevalence increased over time. Government expenditure on medications for pregnant women is rising at a rapid rate in comparison with patient out‐of‐pocket expenditure, with almost one‐third of total Government costs being attributed to only three patented, injectable pharmaceuticals – enoxaparin sodium, ferric carboxymaltose and insulin aspart. The average number of PBS‐listed medications dispensed per pregnancy increased from 2.1 to 2.5 between 2013 and 2018, with an incongruent rise observed for Government expenditure on medication per pregnancy, rising from $46 to $84 over the same time. That is, the rate of increase in Government costs was five times the rate of increase in quantity of items dispensed (per woman).

### 4.2 Interpretation

Other studies that have examined prescription medication use during pregnancy have shown similar prevalence rates, with a Norwegian study^12^ reporting a 60% prevalence and a Danish study^13^ reporting 66%. In terms of therapeutic classes, a 2018 study by Haas et al.^3^ reported findings similar to ours, with gastrointestinal or antiemetic agents, antibiotics and analgesics being the most frequently prescribed therapeutic classes. An Australian pharmacovigilance study based on above co‐payment dispensing data^16^ also reported similar dispensing patterns, with a slightly higher dispensing rate shown for psychoanaleptics. Our study is the first to report on the cost burden of pharmaceuticals and therapeutic classes dispensed during pregnancy. We are only aware of one small study in Brazil that has analysed the costs associated with medication use in 47 pregnant women, reporting only the mean cost of medication per pregnancy (equivalent to $61.83 AUD 2020/21).^2^

We hypothesise that the disproportionate rise in Government expenditure compared with women's out‐of‐pocket expenditure seen in our analysis is influenced by newer, more expensive drugs being prescribed more frequently. Ferric carboxymaltose is an example of this phenomenon, with prescription of this drug increasing approximately five‐fold from 2013 to 2017, and total annual expenditure on intravenous iron therapies for women of reproductive age increasing 35‐fold over the same timeframe.^39^ As the policy landscape changes to accommodate the testing of new and existing medications in pregnant populations, we expect to see newer medications prescribed more frequently, inevitably leading to rises in the mean cost per dispensing in this population. Nonetheless, significant improvements in health outcomes for women and their children are also expected, leading to efficiency gains in the delivery of health care.

Total annual Government expenditure on PBS‐listed medications dispensed during pregnancy increased by more than 50% in real terms from 2013 to 2017. This is twice the increase observed across the entire PBS from 2012/2013 to 2016/2017 (25%).^40^, ^41^ The rate of increase in Government expenditure was also shown to be higher for women who elect private obstetric care as opposed to publicly funded obstetric care. The results also indicate that women who elect privately funded care (versus public) may be prescribed newer, more expensive drugs at a higher rate, where a larger proportion of the cost of medications are borne by the Government. This could indicate equity issues surrounding access to newer medications during pregnancy for women of lower socio‐economic status and warrants further research. Further studies are also required to determine whether the observed increase in expenditure corresponded to improved health outcomes. Such analyses were outside the scope of this paper.

Patient co‐payments for PBS‐listed medications mean that high‐volume medications aren’t always a high cost‐burden to the Government (e.g. metoclopramide). Volume of use, price per dispensing, mode of administration and the availability of generic alternatives all influence the likelihood of a medication being a cost burden to the Government. In addition, multiple other factors influence changes in dispensing patterns and costs over time, including changes in therapeutic guidelines (in particular for diabetes^42^), alterations to pregnancy safety classifications, the inclusion of new medications/new listings on the PBS and the proportion of concessional patients in the community. Investigation of the influence of each of these factors on the results reported were outside the scope of this paper.

Interestingly, ondansetron was shown to be the tenth most frequently dispensed medication despite not being formally approved for use outside of cancer chemotherapy under the funding provisions of the PBS. This may indicate that PBS ‘leakage’ or upward prescribing is a common phenomenon within the antiemetic and antinauseant therapeutic category, whereby medical practitioners prioritise the clinical need of the patient and protection of the doctor–patient relationship over subsidy restrictions.^43^ This observation has been previously reported by Colvin et al.,^44^ who describe the circumstances surrounding these PBS‐funded dispensings in greater detail.

### 4.3 Limitations

The limitations associated with analyses that utilise healthcare databases are well‐known^13^, ^45^ and are applicable to this study. First, there are difficulties in accurately defining medications that were consumed during pregnancy, as we did not have the exact date of delivery (a privacy protection mechanism). Sensitivity analyses showed our primary results were robust to reasonable variations surrounding the assumed date of delivery. Secondly, we acknowledge that the date of dispensing and the date women take medications are not necessarily the same – particularly for drugs used as episodic treatment. For our analyses, we necessarily assumed that when a medication was dispensed it was also taken on the same date, as there was no way of confirming when (or whether) a medication was actually taken. It is also possible that medications may have been dispensed prior to conception, yet taken during pregnancy. Thirdly, drugs may have been prescribed for indications outside of their ATC‐assigned classification (e.g. valproate prescribed for bipolar disorder rather than epilepsy), therefore there may be inaccuracies in the results reported. Fourthly, this study does not report on all medications supplied to women during pregnancy. We did not analyse data relating to:

1. non‐PBS funded prescription medications (including medicines dispensed in a hospital inpatient setting, private prescriptions, over‐the‐counter medications, vitamins or herbal supplements);
2. PBS‐listed items dispensed to pregnant women who experienced a miscarriage or termination of pregnancy prior to 20 weeks of gestation;
3. dispensings relating to pregnancies where the PDC‐PBS link was not successful (*n* = 187);
4. dispensings associated with deliveries where the birth year was unknown (*n* = 93);
5. items dispensed during the first 30 days of pregnancy.

Consequently, it is likely that our analysis underestimates the true prevalence of medication use during pregnancy, volume of dispensings, and total costs associated with PBS‐listed medications. Finally, our analysis has not incorporated an assessment of the incidence of any positive or negative health outcomes linked to consumption of medications; that is, there has been no assessment of the value arising from the medications dispensed. Rather, our analysis serves as a precursor to these types of full economic evaluations, highlighting the therapeutic areas and medications that may require more thorough assessment regarding economic efficiency and cost containment.

## 5 CONCLUSION

Medication use during pregnancy is common and has rising cost implications for women and rapidly escalating cost implications for the Government. Increases in out‐of‐pocket expenses and apparent disparities in access to newer medications between public and private patients reveal issues surrounding equity of access to medications within this population, and warrant further research.

## AUTHOR CONTRIBUTIONS

All authors contributed to conceptualisation of the study. HJ carried out the data analysis for the study under the guidance of EC. HJ drafted the paper, which was edited according to the valuable contributions that all authors made with respect to recommendations for further analysis and interpretation of the data. All authors approved the final version of the article and accept accountability for the integrity of the research.

## FUNDING INFORMATION

HJ was supported by a Monash Equity Scholarship. EC was supported by a National Health and Medical Research Council (NHMRC) Fellowship. LEG was supported by a Channel 7 Children's Research Foundation Fellowship.

## CONFLICT OF INTEREST STATEMENT

EC has received grant funding from Ferring Pharmaceuticals to identify costs associated with adverse birth outcomes of culturally and linguistically diverse women. This funding was not utilised as a part of this study, nor did Ferring Pharmaceuticals play any role in this study. Completed disclosure of interest forms are available to view online as supporting information.

## ETHICS APPROVAL

Ethics approval was obtained from the Townsville Hospital and Health Service Human Research Ethics Committee (HREC; HREC/16/QTHS/223) and the Australian Institute of Health and Welfare HREC (EO2017‐1‐338). In addition, we obtained Public Health Act approval (RD007377) for the study.

## Supporting information


Appendix S1.

Data S1.

## Data Availability

The datasets analysed for this study are not publicly available due to the strict ethics and privacy criteria that govern access to the data repository but are available from the corresponding author on appropriate request.
