# Supplementary material for: Pharmacoepidemiology and costs of medications dispensed during pregnancy: A retrospective population‐based study
Source: BJOG. 2023 Apr 11;130(11):1317–27. doi: 10.1111/1471-0528.17472 (PMC10952169; doi:10.1111/1471-0528.17472)
Supplement: Supplementary file 1 — Appendix S1. [file BJO-130-1317-s001.docx]

**SUPPORTING INFORMATION**

*Supplementary Appendices*

**Pharmacoepidemiology and costs of medications dispensed during pregnancy: A retrospective population-based study**

Hannah Jackson^1^, Luke E Grzeskowiak^2,3^, Joanne Enticott^1^, Emily Callander^1^*

1. Monash Centre for Health Research and Implementation (MCHRI), School of Public health and Preventive Medicine, Monash University, Clayton, Victoria, Australia
2. College of Medicine and Public Health, Flinders University, South Australia, Australia
3. SAHMRI Women and Kids, South Australian Health and Medical Research Institute, South Australia, Australia

** Corresponding author*: [emily.callander@monash.edu](mailto:emily.callander@monash.edu)

**Figure S1** Diagram depicting the variables used to determine whether a dispensing occurred during pregnancy.

A dispensing that occurred during pregnancy is defined as any dispensing that occurred between (but not on) DATE 1 (variable name = start_of_preg_adj) and DATE 2 (variable name = birth_date).


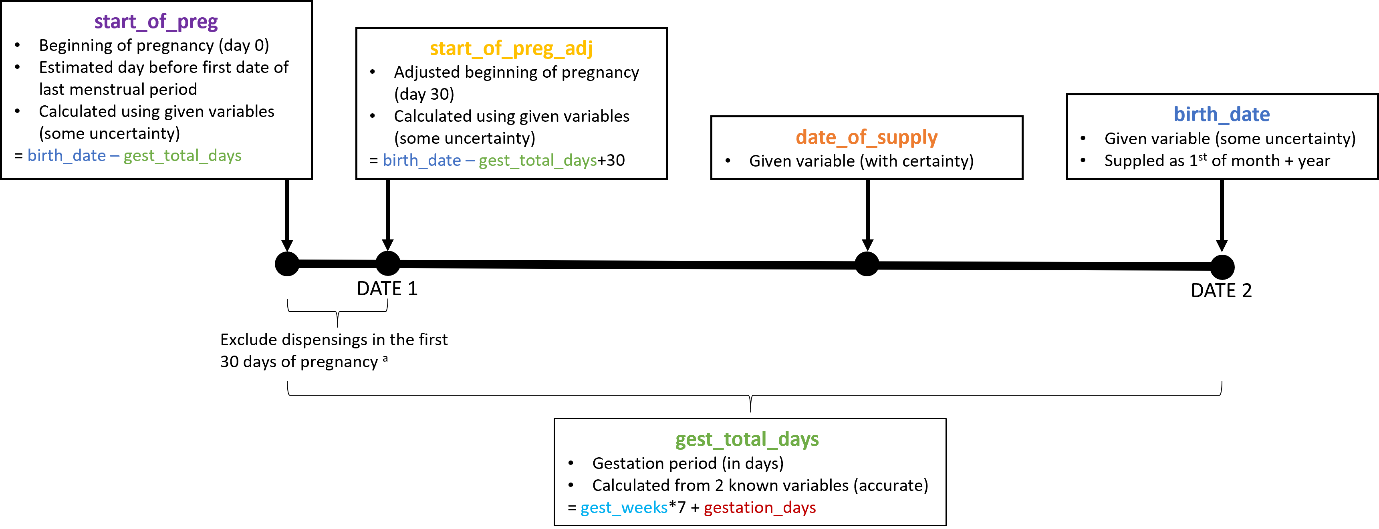


^a^ Average number of days in a month = 365/12=30.42; Mean number of days in the month of delivery within our study population = 30.42. Therefore, we excluded the first 30 days.

**Table S1** GRIPP2-SF checklist

| Section and topic | Item | Reported on page No |
| --- | --- | --- |
| Aim | Report the aim of patient and public involvement in the study | N/A |
| Methods | Provide a clear description of the methods used for patient and public involvement in the study. Please state if there was no patient and public involvement. | No patient or public involvement; reported in methods section of manuscript on page 4. |
| Study results | Outcomes—Report the results of patient and public involvement in the study, including both positive and negative outcomes | N/A |
| Discussion and conclusions | Outcomes—Comment on the extent to which PPI influenced the study overall. Describe positive and negative effects | N/A |
| Reflections/critical perspective | Comment critically on the study, reflecting on the things concerning patient public involvement that went well and those that did not, so others can learn from this experience | N/A |

**Table S2** Characteristics of pregnant women, Queensland, Australia, 2013 to 2018^*^ (incorporating a description of missing data)

| Maternal characteristics | Number (n) | Percentage (%) |  |  |
| --- | --- | --- | --- | --- |
| Plurality  Singleton  Multiple | 323,912  4,956 | 98.49%  1.51% |  |  |
| Maternal age  <20  20 to <35  35 or greater | 9,151  245,202  74,515 | 2.78%  74.56%  22.66% |  |  |
| Gravidity  First pregnancy (primigravida)  Not first pregnancy (multigravida)  Unknown | 100,568  228,297  3 | 30.58%  69.42%  0.00% |  |  |
| Country of birth  Australia  Other  Unknown | 240,515  88,336  17 | 73.13%  26.86%  0.01% |  |  |
| Indigenous status  Yes  No  Unknown | 19,231  309,623  14 | 5.85%  94.15%  0.00% |  |  |
| Funding for antenatal care  Public  Private  Unknown | 226,817  101,372  679 | 68.97%  30.82%  0.21% |  |  |
| Smoking status  Before 20 weeks  Yes  No  Unknown  After 20 weeks  Yes  No  Unknown | 38,865  289,044  959  31,396  296,042  1,430 | 11.82%  87.89%  0.29%  9.55%  90.02%  0.43% |  |  |
| BMI category ^a^  Underweight  Healthy weight  Overweight  Obese  Unknown | 18,196  168,251  74,210  63,700  4,511 | 5.53%  51.16%  22.57%  19.37%  1.37% |  |  |
| Medical conditions ^b^  Yes  No  Unknown | 97,403  231,457  8 | 29.62%  70.38%  0.00% |  |  |
| Pregnancy complication ^c^  Yes  No  Unknown | 230,200  98,661  7 | 70.00%  30.00%  0.00% |  |  |
| Assisted reproductive technology (ART)  Yes  No  Unknown | 17,371  311,489  8 | 5.28%  94.72%  0.00% |  |  |
| Born alive  Yes  No (stillbirth) | 327,278  1,590 | 99.52%  0.48% |  |  |
| Total number of pregnancies | **328,868** | **100%** |  |  |
| ^*^ Data only available for births up until 30^th^ June 2018.  ^a^ BMI calculation uses the self-reported weight of the mother in the 4-6 weeks prior to or at conception.  ^b^ Pre-existing maternal conditions, diseases or illnesses (e.g., hypertension, diabetes), and other conditions, diseases or illnesses that arise during the current pregnancy. Conditions included here are not directly caused by the pregnancy but may influence pregnancy care.  ^c^ Complications of pregnancy that arise prior to the initiation of labour or birth. Conditions included here are directly caused by the pregnancy and may influence pregnancy care. | | |  |  |

**Figure S2** Demographic trends for pregnant women, Queensland, Australia, 2013 to 2018^*^

| 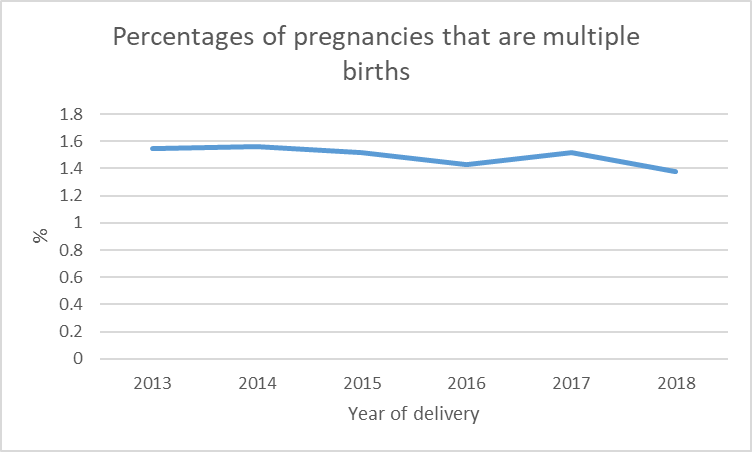 | 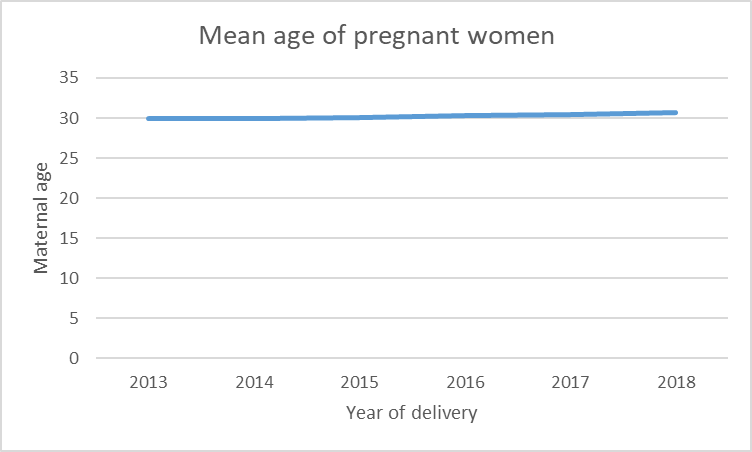 | 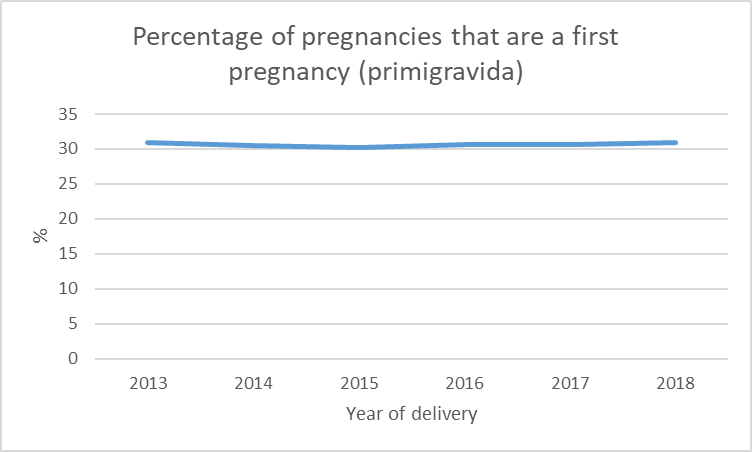 | 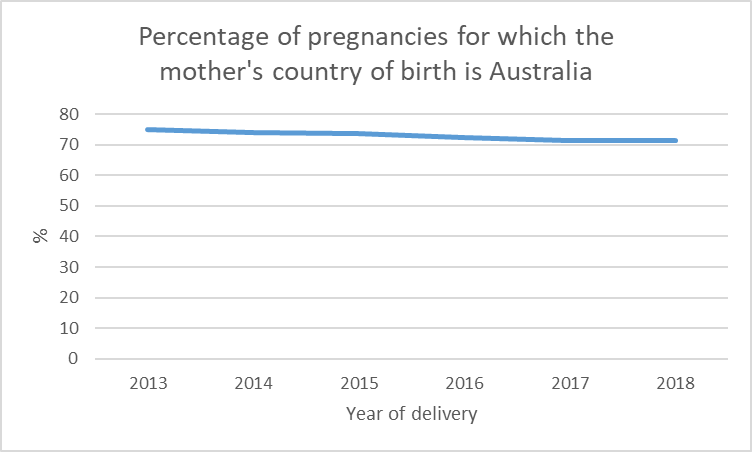 |
| --- | --- | --- | --- |
| 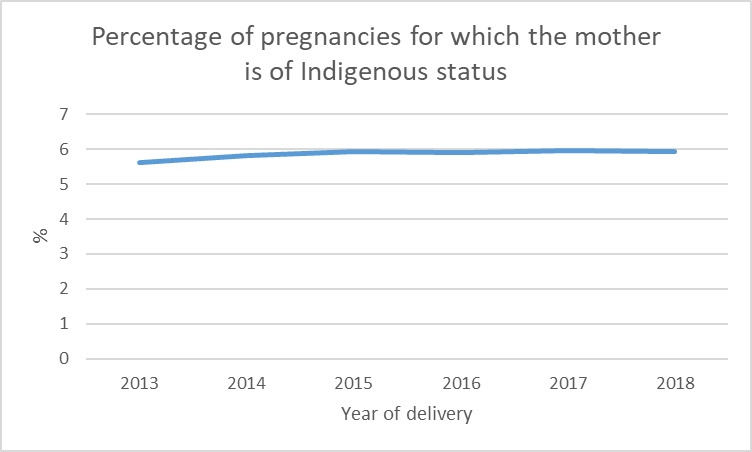 |  | 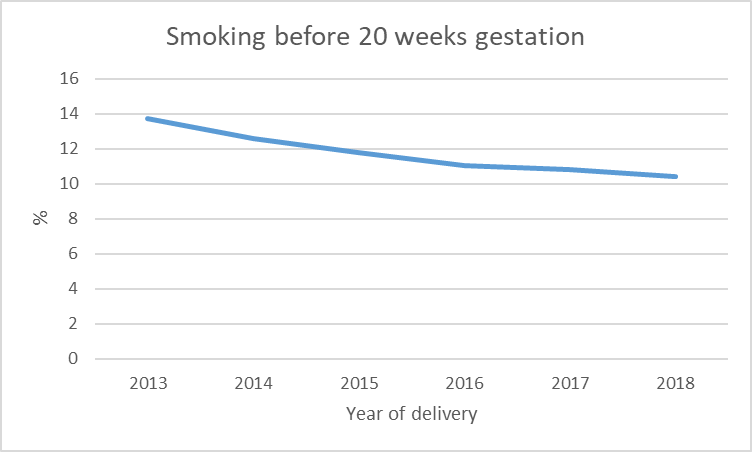 | 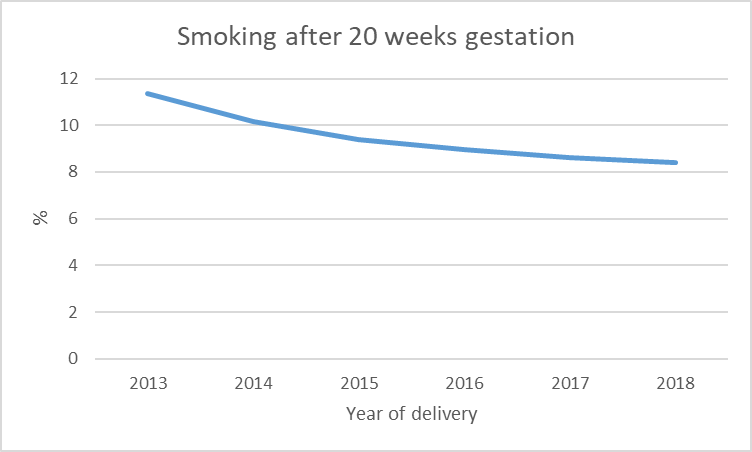 |
| 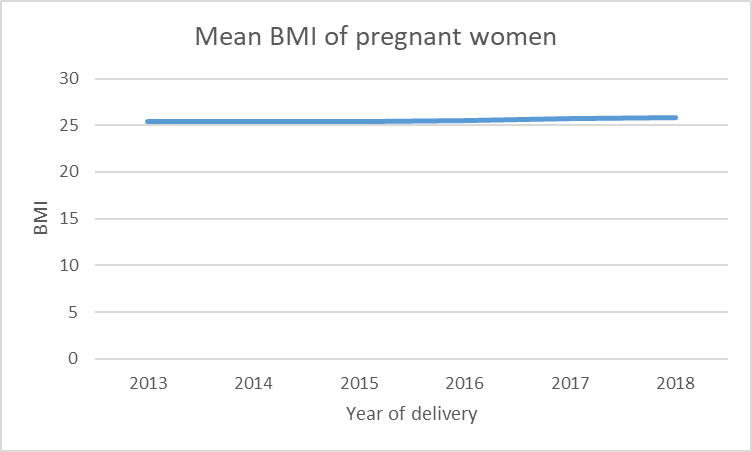 | 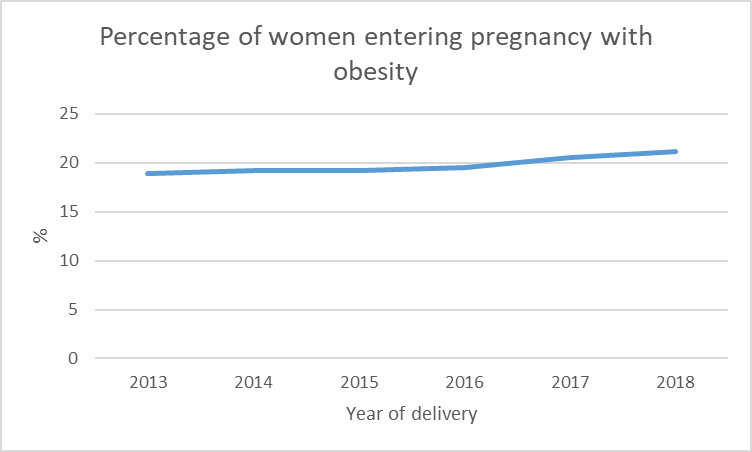 | 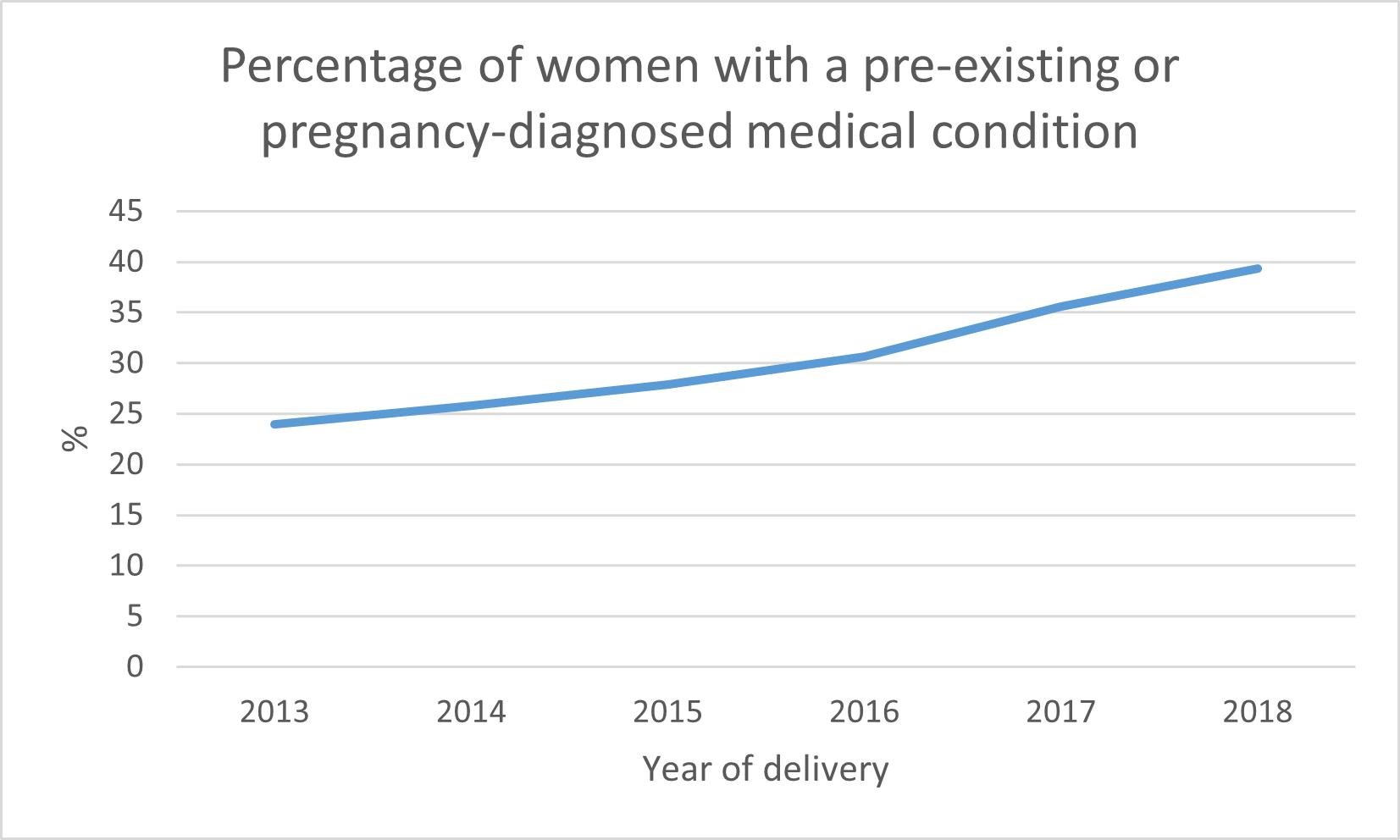 | 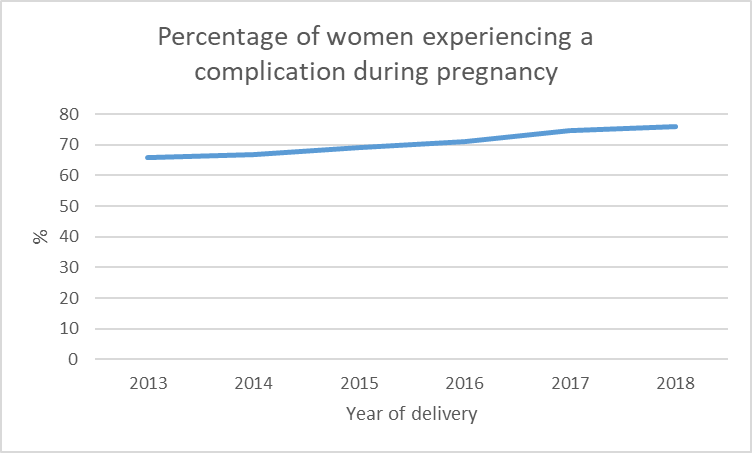 |
| 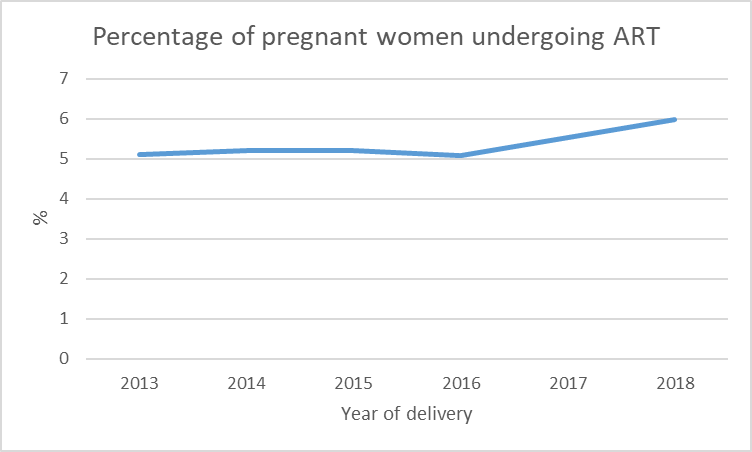 | 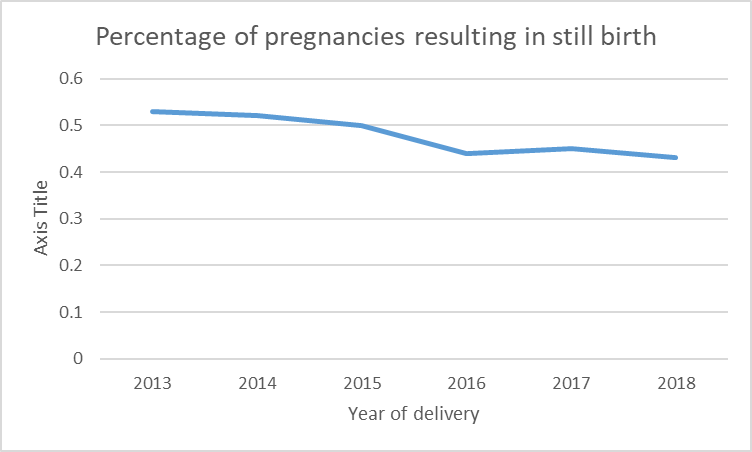 |  |  |

^*^ Data only available for births up until 30^th^ June 2018

**Table S3** Number and percentage of pregnant women using medication approved for public subsidy over time, Queensland, Australia, 2013 to 2018^*^

|  | Year of delivery | | | | | | |
| --- | --- | --- | --- | --- | --- | --- | --- |
|  | **Overall** | **2013** | **2014** | **2015** | **2016** | **2017** | **2018** |
| Entire pregnancy  N, %  (95% CI) | 201,022  61.13%  (60.96, 61.29) | 35,858  58.39%  (58.00, 58.78) | 36,644  60.03%  (59.64, 60.42) | 36,737  61.45%  (61.06, 61.84) | 37,234  62.07%  (61.68, 62.46) | 36,064  62.75%  (62.35, 63.14) | 18,485  63.36%  (62.81, 63.92) |
| 1^st^ trimester  N, %  (95% CI) | 122,124  37.13%  (36.97, 37.30) | 21,314  34.71%  (34.33, 35.08) | 21,906  35.89%  (35.51, 36.27) | 22,310  37.32%  (36.93, 37.71) | 22,954  38.26%  (37.88, 38.65) | 22,135  38.51%  (38.11, 38.91) | 11,505  39.44%  (38.88, 40.00) |
| 2^nd^ trimester  N, %  (95% CI) | 112,239  34.13%  (33.97, 34.29) | 20,115  32.76%  (32.38, 33.13) | 20,483  33.56%  (33.18, 33.93) | 20,644  34.53%  (34.15, 34.91) | 20,758  34.60%  (34.22, 34.98) | 19,890  34.61%  (34.22, 34.99) | 10,349  35.47%  (34.93, 36.02) |
| 3^rd^ trimester  N, %  (95% CI) | 109,502  33.30%  (33.14, 33.46) | 18569  30.24%  (29.88, 30.60) | 19,590  32.09%  (31.72, 32.46) | 20,069  33.57%  (33.19, 33.95) | 20,369  33.96%  (33.58, 34.33) | 20,507  35.68%  (35.29, 36.07) | 10,398  35.64%  (35.09, 36.19) |
| Abbreviation:  95% CI = 95% confidence interval | | | | | | | |

^*^ Data only available for births up until 30^th^ June 2018

**Table S4** Total cost to either pregnant women (patient out-of-pocket (OOP) expenditure) or the Government (PBS expenditure) according to the ATC Classification System (Level 2), Queensland, Australia, 2013 to 2018^*^, in constant prices (AUD 2020/21)

| Total cost to pregnant women (total OOP expenditure) | | | | | Total Government expenditure (PBS expenditure) | | | | |
| --- | --- | --- | --- | --- | --- | --- | --- | --- | --- |
| ATC Classification  (ATC code, level 2) | **Number of dispensings** | **Total patient expenditure per class** | **Percent of total patient expenditure** | **Cumulative percent** | **ATC Classification**  **(ATC code, level 2)** | **Number of dispensings** | **Government expenditure** | **Percent of total Government expenditure** | **Cumulative percent** |
| 1. Antibacterials for systemic use (J01) | 199,496 | $2,037,191.28 | 18.17 | 18.17 | 1. Drugs used in diabetes (A10) | 39,250 | $3,394,343.25 | 17.56 | 17.56 |
| 2. Psychoanaleptics (N06) | 86,597 | $1,239,083.11 | 11.05 | 29.22 | 2. Antithrombotic agents (B01) | 22,043 | $2,624,096.85 | 13.57 | 31.13 |
| 3. Drugs for obstructive airway diseases (R03) | 41,071 | $811,075.46 | 7.23 | 36.46 | 3. Immunosuppressants (L04) | 2,679 | $2,604,406.84 | 13.47 | 44.61 |
| 4. Drugs for functional gastrointestinal disorders (A03) | 84,982 | $790,522.88 | 7.05 | 43.51 | 4. Antianaemic preparations (B03) | 22,789 | $2,222,176.07 | 11.50 | 56.10 |
| 5. Drugs for acid related disorders (A02) | 49,977 | $782,124.22 | 6.98 | 50.49 | 5. Antivirals for systemic use (J05) | 6,385 | $1,257,905.28 | 6.51 | 62.61 |
| 6. Drugs used in diabetes (A10) | 39,250 | $750,210.40 | 6.69 | 57.18 | 6. Sex hormones and modulators of the genital system (G03) | 21,059 | $932,471.09 | 4.82 | 67.43 |
| 7. Antithrombotic agents (B01) | 22,043 | $721,842.55 | 6.44 | 63.62 | 7. Drugs for obstructive airway diseases (R03) | 41,071 | $930,106.03 | 4.81 | 72.24 |
| 8. Thyroid therapy (H03) | 21,109 | $511,479.96 | 4.56 | 68.18 | 8. Antidiarrheals, intestinal antiinflammatory/antiinfective (A07) | 4,399 | $625,029.00 | 3.23 | 75.48 |
| 9. Sex hormones and modulators of the genital system (G03) | 21,059 | $485,162.20 | 4.33 | 72.51 | 9. Psychoanaleptics (N06) | 86,597 | $620,299.85 | 3.21 | 78.69 |
| 10. Analgesics (N02) | 44,124 | $425,141.59 | 3.79 | 76.30 | 10. Antibacterials for systemic use (J01) | 199,496 | $544,846.50 | 2.82 | 81.50 |
| 11. Antianaemic preparations (B03) | 22,789 | $378,175.30 | 3.37 | 79.67 | 11. Psycholeptics (N05) | 15,831 | $535,423.71 | 2.77 | 84.27 |
| 12. Antiemetics and antinauseants (A04) | 22,418 | $368,594.21 | 3.29 | 82.96 | 12. Analgesics (N02) | 44,124 | $330,820.98 | 1.71 | 85.99 |
| 13. Corticosteroids, dermatological preparations (D07) | 15,611 | $222,092.91 | 1.98 | 84.94 | 13. General nutrients (V06) | 241 | $316,290.03 | 1.64 | 87.62 |
| 14. Antivirals for systemic use (J05) | 6,385 | $199,599.16 | 1.78 | 86.72 | 14. Antiepileptics (N03) | 8,704 | $264,312.29 | 1.37 | 88.99 |
| 15. Antiepileptics (N03) | 8,704 | $196,613.77 | 1.75 | 88.48 | 15. Other respiratory system products (R07) | 10 | $237,949.12 | 1.23 | 90.22 |
| 16. Corticosteroids for systemic use (H02) | 14,225 | $181,512.42 | 1.62 | 90.10 | 16. Immunostimulants (L03) | 193 | $222,994.23 | 1.15 | 91.37 |
| 17. Psycholeptics (N05) | 15,831 | $147,291.34 | 1.31 | 91.41 | 17. Drugs for acid related disorders (A02) | 49,977 | $189,964.92 | 0.98 | 92.36 |
| 18. Antidiarrheals, intestinal antiinflammatory/antiinfective (A07) | 4,399 | $144,787.07 | 1.29 | 92.70 | 18. Antiemetics and antinauseants (A04) | 22,418 | $177,183.63 | 0.92 | 93.27 |
| 19. Antihypertensives (C02) | 8,204 | $128,171.18 | 1.14 | 93.85 | 19. Antineoplastic agents (L01) | 556 | $157,589.23 | 0.82 | 94.09 |
| 20. Immunosuppressants (L04) | 2,679 | $ 92,316.43 | 0.82 | 94.67 | 20. Drugs for functional gastrointestinal disorders (A03) | 84,982 | $135,485.90 | 0.70 | 94.79 |

^*^ Data only available for births up until 30^th^ June 2018

**Figure S3** Subgroup analysis showing the public versus private volume and cost of PBS-listed medications dispensed during pregnancy from the perspective of both patients and the Government over time, Queensland, Australia, 2013 to 2018^*^, in constant prices (AUD 2020/21)

| 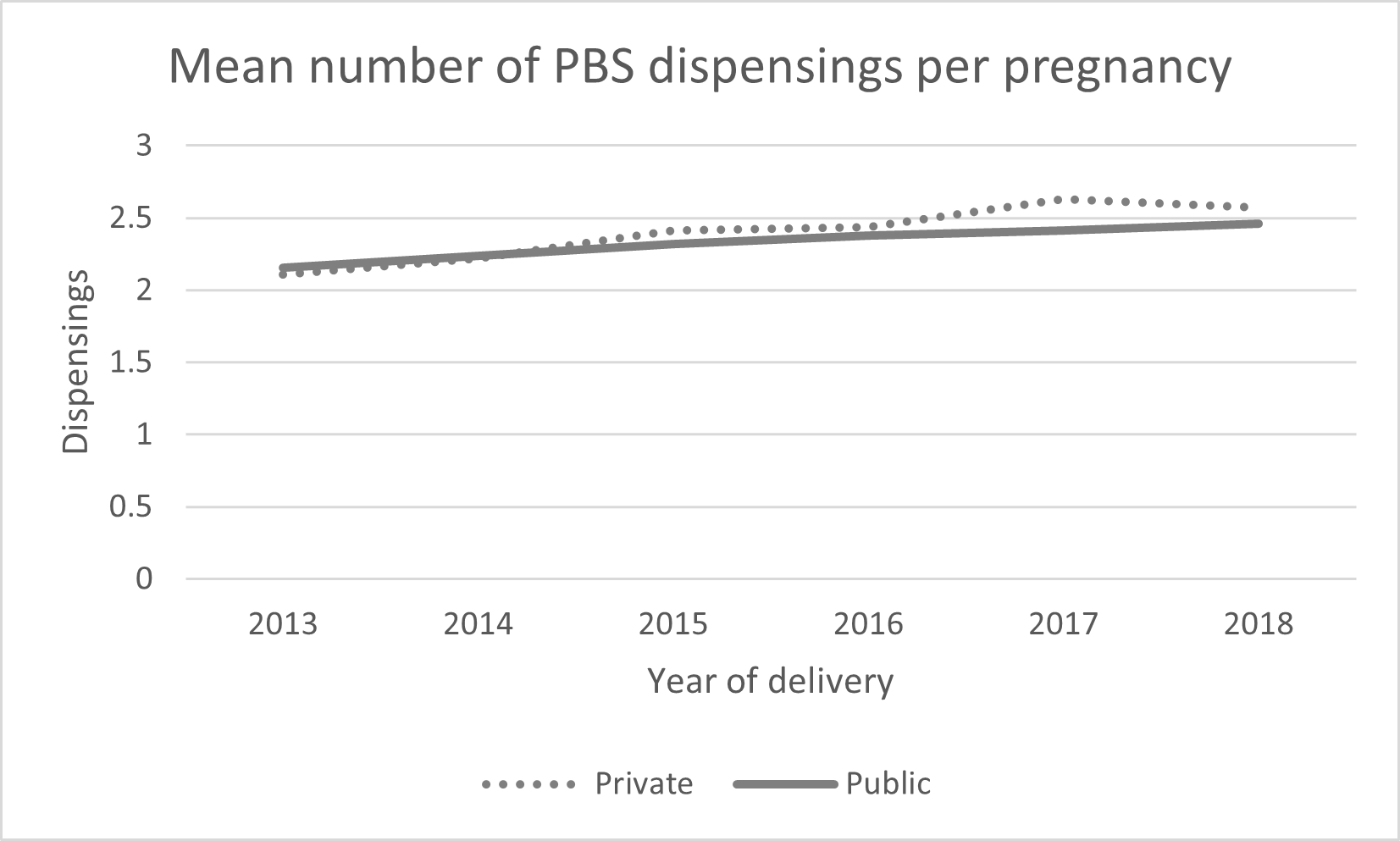 | |
| --- | --- |
| 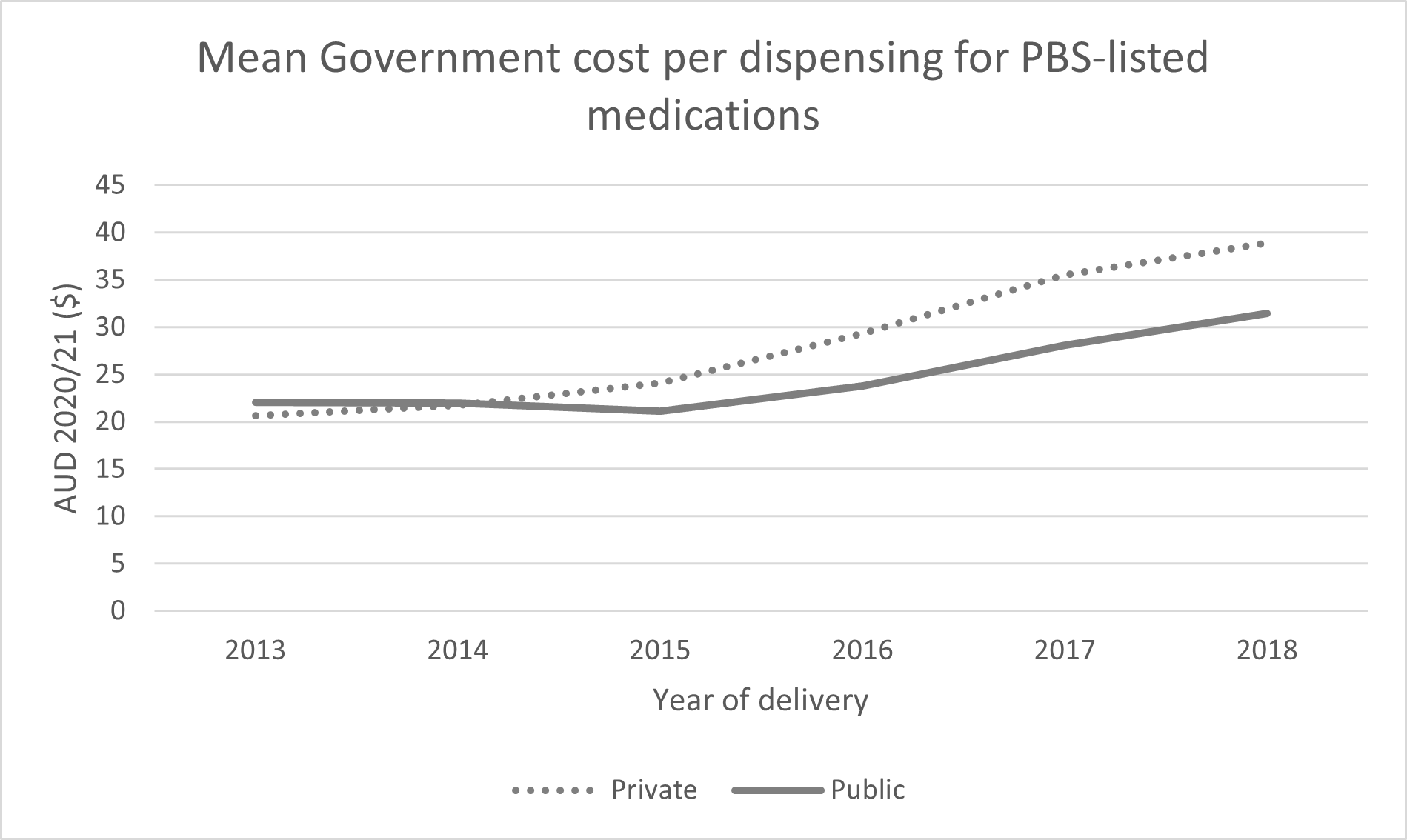  *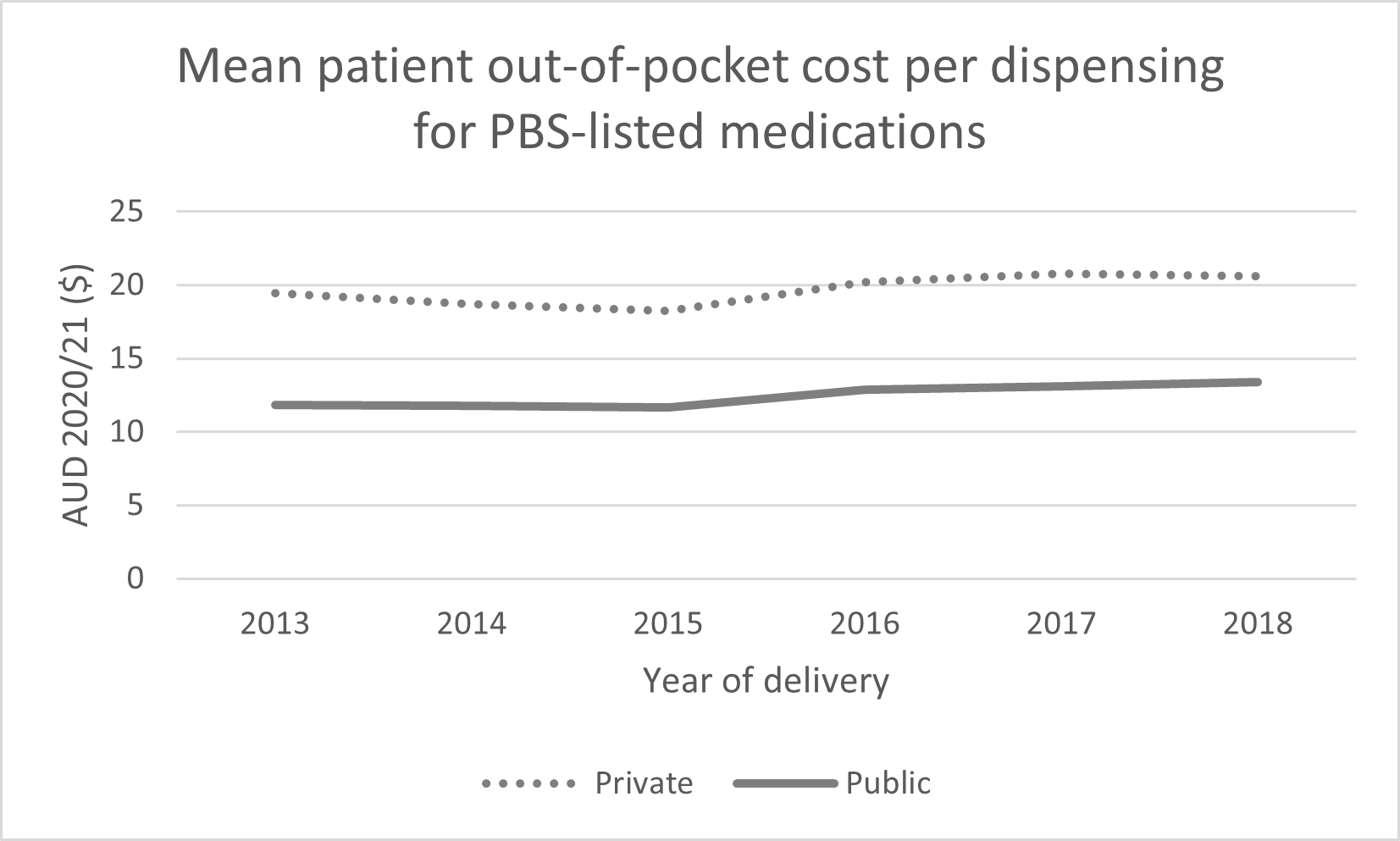* | 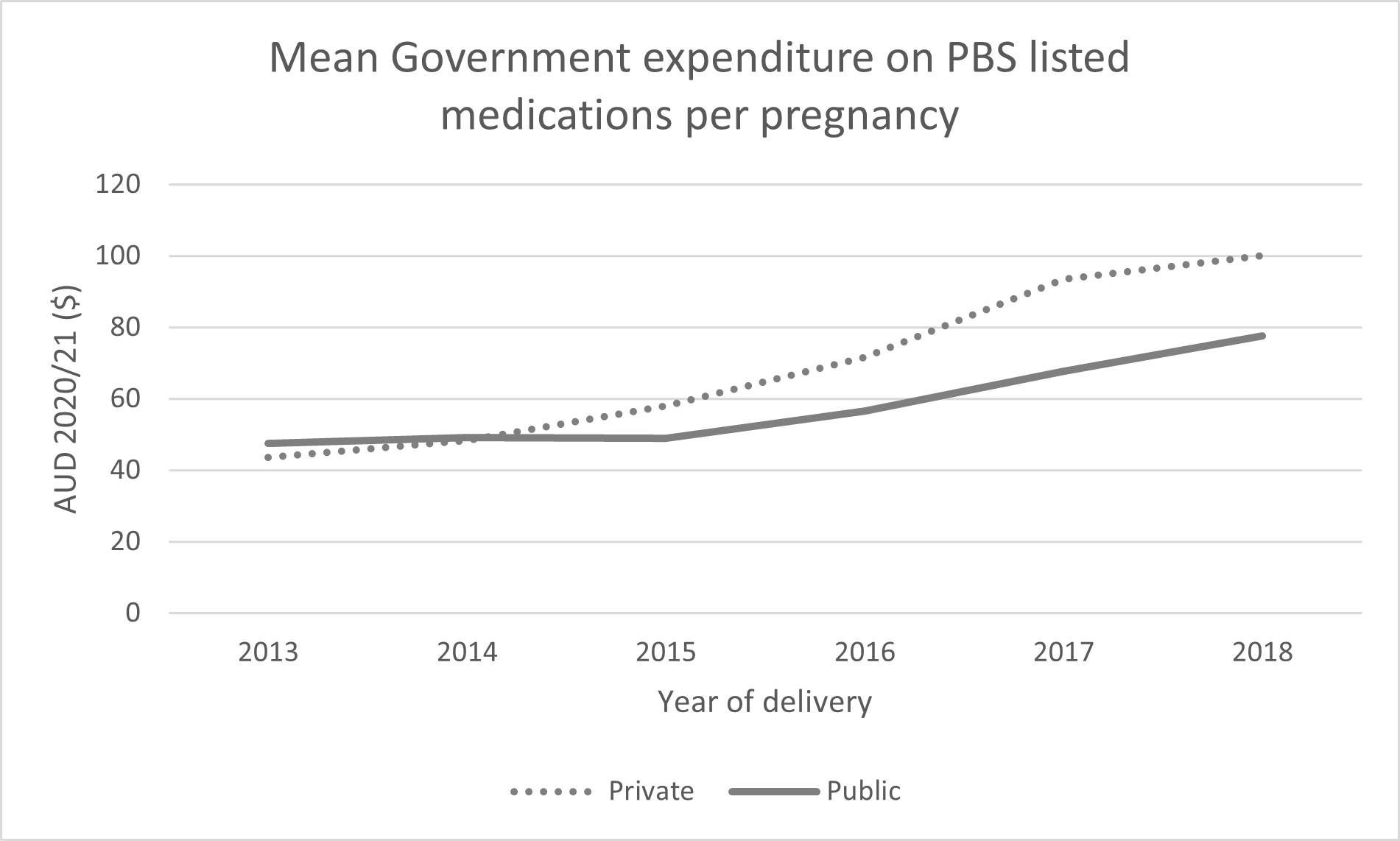  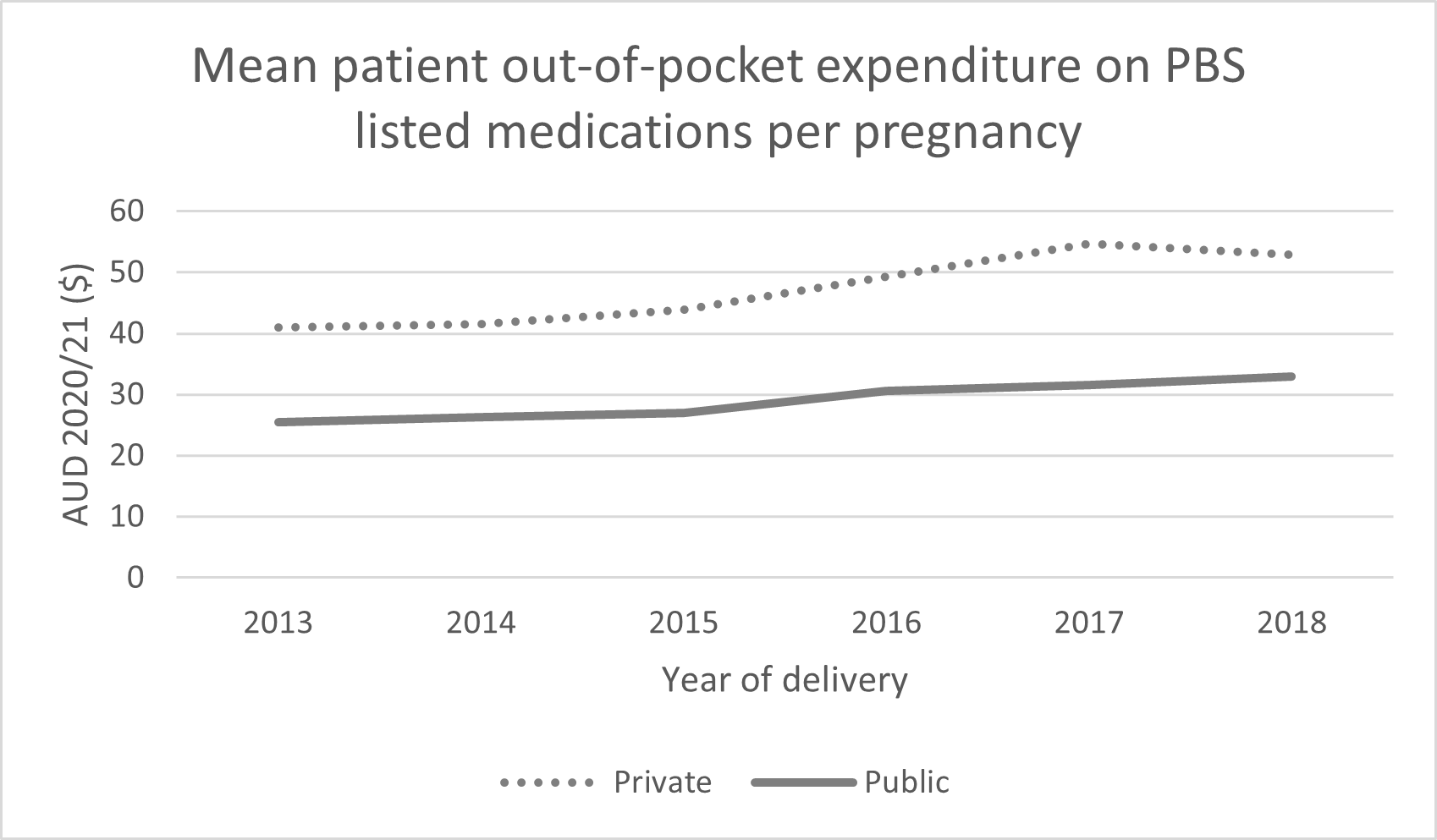 |
|  |  |

^*^ Data only available for births up until 30^th^ June 2018

**Table S5** Sensitivity analysis for volume of pharmaceuticals dispensed during pregnancy using alternative assumptions for date of delivery

|  | **PRIMARY ANALYSIS (1ST OF BIRTH MONTH)** | | | | **SENSITIVITY ANALYSIS (15TH OF BIRTH MONTH)** | | | | **SENSITIVITY ANALYSIS (END OF BIRTH MONTH)** | | | |
| --- | --- | --- | --- | --- | --- | --- | --- | --- | --- | --- | --- | --- |
|  | Pharmaceutical agent | N | % | Cum. % | Pharmaceutical agent | N | % | Cum. % | Pharmaceutical agent | N | % | Cum. % |
| 1. | Metoclopramide | 84504 | 11 | 11.04 | Metoclopramide | 83418 | 10.19 | 10.19 | Cefalexin | 92254 | 10.25 | 10.25 |
| 2. | Amoxicillin | 72889 | 9.52 | 20.55 | Cefalexin | 78911 | 9.64 | 19.83 | Amoxicillin | 74272 | 8.25 | 18.49 |
| 3. | Cefalexin | 72664 | 9.49 | 30.04 | Amoxicillin | 74205 | 9.06 | 28.9 | Metoclopramide | 72202 | 8.02 | 26.51 |
| 4. | Sertraline | 25380 | 3.31 | 33.36 | Paracetamol + codeine | 25879 | 3.16 | 32.06 | Oxycodone | 43771 | 4.86 | 31.37 |
| 5. | Paracetamol + codeine | 24438 | 3.19 | 36.55 | Sertraline | 25414 | 3.1 | 35.16 | Enoxaparin sodium | 28594 | 3.18 | 34.55 |
| 6. | Metformin | 23429 | 3.06 | 39.61 | Metformin | 24697 | 3.02 | 38.18 | Paracetamol + codeine | 27941 | 3.1 | 37.65 |
| 7. | Ranitidine | 21919 | 2.86 | 42.47 | Enoxaparin sodium | 24319 | 2.97 | 41.15 | Sertraline | 25977 | 2.88 | 40.54 |
| 8. | Enoxaparin sodium | 20864 | 2.72 | 45.2 | Ranitidine | 23873 | 2.92 | 44.07 | Metformin | 24795 | 2.75 | 43.29 |
| 9. | Levothyroxine | 20040 | 2.62 | 47.81 | Oxycodone | 22285 | 2.72 | 46.79 | Ranitidine | 24338 | 2.7 | 45.99 |
| 10. | Ondansetron | 19986 | 2.61 | 50.42 | Ondansetron | 20070 | 2.45 | 49.24 | Amoxicillin + clavulanic acid | 23442 | 2.6 | 48.6 |
| 11. | Salbutamol | 18427 | 2.41 | 52.83 | Levothyroxine | 19988 | 2.44 | 51.68 | Levothyroxine | 18975 | 2.11 | 50.7 |
| 12. | Escitalopram | 15469 | 2.02 | 54.85 | Salbutamol | 18403 | 2.25 | 53.93 | Ondansetron | 18621 | 2.07 | 52.77 |
| 13. | Amoxicillin + clavulanic acid | 13282 | 1.73 | 56.58 | Amoxicillin + clavulanic acid | 15905 | 1.94 | 55.87 | Salbutamol | 18142 | 2.01 | 54.79 |
| 14. | Venlafaxine | 10918 | 1.43 | 58.01 | Escitalopram | 15071 | 1.84 | 57.71 | Escitalopram | 15103 | 1.68 | 56.46 |
| 15. | Rabeprazole | 10722 | 1.4 | 59.41 | Rabeprazole | 11544 | 1.41 | 59.12 | Tramadol | 13652 | 1.52 | 57.98 |
| 16. | Fluticasone propionate + salmeterol | 9914 | 1.29 | 60.7 | Venlafaxine | 10638 | 1.3 | 60.42 | Metronidazole | 12380 | 1.37 | 59.36 |
| 17. | Estradiol | 9598 | 1.25 | 61.96 | Fluticasone propionate + salmeterol | 9828 | 1.2 | 61.62 | Rabeprazole | 11771 | 1.31 | 60.66 |
| 18. | Erythromycin ethylsuccinate | 8627 | 1.13 | 63.08 | Ferric carboxymaltose | 9326 | 1.14 | 62.76 | Ferric carboxymaltose | 10516 | 1.17 | 61.83 |
| 19. | Desvenlafaxine | 8447 | 1.1 | 64.19 | Methyldopa | 8891 | 1.09 | 63.85 | Venlafaxine | 10405 | 1.16 | 62.99 |
| 20. | Oxycodone | 8145 | 1.06 | 65.25 | Erythromycin ethylsuccinate | 8650 | 1.06 | 64.91 | Fluticasone propionate + salmeterol | 9676 | 1.07 | 64.06 |

**Table S6** Sensitivity analysis for total Government expenditure (AUD 2020/21) on pharmaceuticals dispensed to pregnant women using alternative assumptions for date of delivery

|  | **PRIMARY ANALYSIS (1ST OF BIRTH MONTH)** | | | | **SENSITIVITY ANALYSIS (15TH OF BIRTH MONTH)** | | | | **SENSITIVITY ANALYSIS (END OF BIRTH MONTH)** | | | |
| --- | --- | --- | --- | --- | --- | --- | --- | --- | --- | --- | --- | --- |
|  | Pharmaceutical agent | N | Gov't cost | % | Pharmaceutical agent | N | Gov't cost | % | Pharmaceutical agent | N | Gov't cost | % |
| 1. | Enoxaparin sodium | 20,864 | $2,581,818.81 | 13.36 | Enoxaparin sodium | 24,319 | $2,758,369.25 | 14.12 | Ferric carboxymaltose | 10,516 | $2,937,770.34 | 14.51 |
| 2. | Ferric carboxymaltose | 7,301 | $2,041,785.79 | 10.56 | Ferric carboxymaltose | 9,326 | $2,608,819.73 | 13.35 | Enoxaparin sodium | 28,594 | $2,930,332.96 | 14.47 |
| 3. | Insulin aspart | 5,684 | $1,155,155.55 | 5.98 | Insulin aspart | 6,106 | $1,219,482.73 | 6.24 | Insulin aspart | 6,165 | $1,221,572.23 | 6.03 |
| 4. | Adalimumab | 591 | $1,076,963.84 | 5.57 | Adalimumab | 553 | $1,005,530.27 | 5.15 | Adalimumab | 531 | $963,256.87 | 4.76 |
| 5. | Insulin isophane human | 5,797 | $785,529.93 | 4.06 | Insulin isophane human | 6,176 | $825,139.21 | 4.22 | Insulin isophane human | 6,231 | $826,457.62 | 4.08 |
| 6. | Insulin glargine | 1,507 | $585,211.54 | 3.03 | Insulin glargine | 1,595 | $612,516.37 | 3.14 | Insulin glargine | 1,587 | $602,617.97 | 2.98 |
| 7. | Mesalazine | 2,128 | $572,384.95 | 2.96 | Mesalazine | 2,139 | $572,802.48 | 2.93 | Mesalazine | 2,140 | $574,154.48 | 2.84 |
| 8. | Insulin detemir | 1,435 | $543,545.13 | 2.81 | Insulin detemir | 1,507 | $565,570.59 | 2.90 | Insulin detemir | 1,508 | $561,988.77 | 2.78 |
| 9. | Infliximab | 177 | $522,963.85 | 2.71 | Infliximab | 170 | $501,221.46 | 2.57 | Infliximab | 166 | $487,649.95 | 2.41 |
| 10. | Fluticasone propionate + salmeterol | 9,914 | $445,426.25 | 2.30 | Fluticasone propionate + salmeterol | 9,828 | $439,193.38 | 2.25 | Fluticasone propionate + salmeterol | 9,676 | $431,188.52 | 2.13 |
| 11. | Etanercept | 239 | $404,147.57 | 2.09 | Etanercept | 229 | $385,652.18 | 1.97 | Etonogestrel | 1,894 | $375,223.23 | 1.85 |
| 12. | Tenofovir disoproxil | 353 | $341,636.27 | 1.77 | Tenofovir disoproxil | 373 | $357,766.10 | 1.83 | Tenofovir disoproxil | 391 | $374,767.75 | 1.85 |
| 13. | Follitropin alfa | 248 | $337,385.50 | 1.75 | Amino acid formula with vitamins and minerals without phenylalanine | 144 | $264,409.76 | 1.35 | Etanercept | 218 | $362,268.55 | 1.79 |
| 14. | Amino acid formula with vitamins and minerals without phenylalanine | 140 | $258,008.44 | 1.33 | Budesonide + formoterol (eformoterol) | 5,997 | $243,211.00 | 1.24 | Amino acid formula with vitamins and minerals without phenylalanine | 140 | $256,240.98 | 1.27 |
| 15. | Budesonide + formoterol (eformoterol) | 6,069 | $246,651.79 | 1.28 | Ivacaftor | 8 | $189,396.95 | 0.97 | Budesonide + formoterol (eformoterol) | 5,878 | $237,464.27 | 1.17 |
| 16. | Ivacaftor | 10 | $237,949.12 | 1.23 | Desvenlafaxine | 8,095 | $180,283.87 | 0.92 | Ivacaftor | 8 | $189,396.95 | 0.94 |
| 17. | Progesterone | 1,034 | $206,098.80 | 1.07 | Quetiapine | 2,751 | $172,249.11 | 0.88 | Desvenlafaxine | 7,916 | $174,256.59 | 0.86 |
| 18. | Desvenlafaxine | 8,447 | $189,315.49 | 0.98 | Ondansetron | 20,070 | $169,633.27 | 0.87 | Quetiapine | 2,713 | $167,156.86 | 0.83 |
| 19. | Quetiapine | 2,832 | $180,324.62 | 0.93 | Valaciclovir | 3,230 | $167,675.74 | 0.86 | Valaciclovir | 3,334 | $167,014.27 | 0.82 |
| 20. | Valaciclovir | 3,049 | $168,395.56 | 0.87 | Cefalexin | 78,911 | $149,601.66 | 0.77 | Cefalexin | 92,254 | $162,419.75 | 0.80 |
